# Spatial Zoning of Carbon Dioxide Emissions at the Intra-City Level: A Case Study of Nanjing, China

**DOI:** 10.3390/ijerph20054023

**Published:** 2023-02-23

**Authors:** Yuan Yuan, Ping Xu, Hui Zhang

**Affiliations:** 1 School of Public Administration, Hohai University, Nanjing 211100, China; 2 Department of Land Planning, China Land Surveying and Planning Institute, Beijing 100035, China

**Keywords:** urbanization and carbon neutrality, CHRED, spatial scale transformation, carbon sinks and sources, energy consumption, territorial spatial planning

## Abstract

With ever-increasing urbanization and industrialization in developing countries, the challenge posed by carbon dioxide emissions (CDEs) has become a hot topic of concern in the realm of sustainable development from a socioeconomic perspective. However, previous studies have only been conducted at macro and meso scales, including at the global, country, and urban levels, and few researchers have delved into the territorial space of urban areas due to a lack of high-precision data. To address this deficiency, we established a theoretical framework to explore the spatial zoning of CDEs based on the newly emerging China high-resolution emission gridded data (CHRED). This study’s innovativeness lies in its provision of a step-by-step process for spatial matching of CDEs based on CHRED in the framework and the construction of square layers to reveal spatial heterogeneity of CDEs at the intra-city level. Taking Nanjing City as the case study area, our findings indicated that CDEs intensity (CDEI) shows an inverted “U-shaped” trend that first increased and then decreased, and finally stabilized from the center to the periphery of the city. With further urbanization and industrialization, the energy consumption sector was found to be the largest contributor to CDEs in Nanjing, and the expanding carbon source zonings will therefore shrink the existing carbon sink zonings. Collectively, these results can provide a scientific reference point to realize China’s “dual carbon” target from the perspective of spatial layout optimization.

## 1. Introduction

The latest Greenhouse Gas Bulletin released by the World Meteorological Organization stated that the global concentration of carbon dioxide emissions (CDEs) in the atmosphere had reached a new historical record of 413.2 ppm in 2020 [1]. The sharp rise in CDEs as a result of the human consumption of fossil fuels poses a threat to sustainable human development and ecological environment [2]. In fact, the International Energy Agency (IEA) has estimated that urban areas, which are a gathering place for commercial trade and production factor resources [3], consume 67% of all energy produced globally and account for 75% of worldwide CDEs [4]. Therefore, it is of great importance that accurate estimates of CDEs and their determinants in large urban agglomerations are carried out.

With the increasing emphasis on the effects of climate change and actions to mitigate them in various countries, studies of CDEs cover all scales from macro to micro geographic space, such as global [5], country [6,7], provincial [8,9], city [10], sectoral [11,12], and community scales [13]. Large-scale studies are suited to grasping the overall situation of CDEs from a macro perspective, while the meso and micro perspectives are beneficial for the implementation of effective policies [14]. In fact, urban areas are a classic example of small-scale cases yielding diverse data that require the precise implementation of emissions reduction measures. Many studies have analyzed the relationship between various elements of cities and CDEs. Currently, most urbanized regions, especially those in developing countries, are in the process of continuous migration and transformation, arising from their ongoing industrialization, urbanization, and modernization processes. Studies have shown that there is an inevitable relationship between urbanization and CDEs [5]. Obviously, urbanization results in changes in urban land use (e.g., the constant expansion of land for construction leads to reductions in carbon sinks and the expansion of carbon sources [2]) and spatial patterns (e.g., the diversity, connectivity, and compactness of the urban form affect the type and quantity of air pollutants emitted by different sectors of cities [15,16,17]), which affects the quantity of CDEs to a certain extent, including carbon sinks and sources. Moreover, with the continuous acceleration of urban development, numerous factors have been highlighted in studies of CDEs, including economic growth [18], gross domestic product (GDP) [19], population size and density [20,21], energy structure and consumption [22,23], industrial structure [24], technology [25], foreign direct investment [26], and tourism [27], which are widely recognized as being key factors affecting CDEs. Studies of these factors and CDEs have shown that the latter have significant spatial autocorrelations that display spatial agglomeration effects [28].

Although numerous studies have analyzed the impact of land-use changes on CDEs in different regions and at different scales [29,30,31], it is still very difficult to obtain specific micro scale gridded data on CDEs at the intra-city level. The bottom-up spatial gridded method using population spatial data or other auxiliary data to calculate CDEs was the first to be used by researchers. Then, with the increasing demand for atmospheric model accuracy and satellite remote sensing monitoring capabilities, the Emissions Database for Global Atmospheric Research (EDGAR) was the first to create a spatial gridded database systemically and consistently covering all of the reporting categories in the Intergovernmental Panel on Climate Change (IPCC) guidelines [32,33], with the exception of land use, land use change, and forestry [34]. The EDGAR database compiles IEA emission point data sources and energy consumption data on the basis of independent global sets of activity data, and its time series are updated every year, subject to the availability of their data sources [35]. Therefore, in such a rapidly evolving and complex context, EDGAR has become a reference dataset supporting policy makers and the scientific community [31], providing a reliable and consistent benchmark for studies of CDE calculations [36]. Moreover, the Vulcan inventory has quantified fossil fuel CDEs, which, in 2012, were produced mainly by US sectors using different combustion technologies and 48 fuel types [37]. The Vulcan inventory, which compiles 10 km gridded emissions data, builds on decades of regional air pollution monitoring and complements these data with a Census, Transport and Digital Roads dataset and plays an important role in CDEs research and government decision-making [33]. Recently, high-resolution gridded data and the spatial stratified heterogeneity of CDEs has become a hot topic of emissions research. The derivation of ground CDEs data based on satellite remote sensing data has become an important research field. At present, there are three main satellites monitoring carbon concentrations [38]: GOSAT at 10 × 10 km resolution [39], OCO-2 at 1 × 1 km resolution [40], and Tan Sat at 2 × 2 km resolution [41]. In addition, it is worth noting that, with the development of remote sensing technology, the use of night-time light (NTL) data to estimate gridded CDEs has become an important research direction. Currently, NTL data are primarily obtained by the Defense Meteorological Satellite Program-Operational Line Scan System (DMSP-OLS) and reflect socioeconomic activities from four dimensions: population, economy, society, and space [42]. Thus, the NTL data can provide rich distribution information about socioeconomic activities from multiple dimensions to compensate for the one-dimensional and “no spatial information” deficiencies of studies of the relationship between urban spatial development patterns and CDEs.

China is known as the world’s largest developing country and the leading emitter of carbon dioxide [43], making it a key player in achieving global carbon neutrality. The Chinese government pledged at the 21st United Nations Climate Change Conference held in Paris in 2015 to lower CDEs intensity (CDEI) by 60–65% by 2030 compared to 2005 levels [44]. At the General Debate of the 75th Session of the United Nations General Assembly in 2020, the Chinese government further stated explicitly that CDEs would peak by 2030 and that the government would strive to achieve carbon neutrality by 2060. Therefore, how to obtain accurate calculations of CDEs and promote the implementation of emissions reduction strategies via spatial planning at the intra-city level are undoubtedly important for achieving this goal. Thus, as the basis for obtaining detailed and accurate results for CDEs, high-resolution gridded data play an increasingly important role in examining the spatial differences and their main driving factors in Chinese cities. However, China is still short of high-resolution gridded data. The existing EDGAR gridded data [45], the Oak Ridge National Laboratory (ORNL) CDEs gridded data [46], and the Peking University (PKU) inventory [47] estimate China’s gridded emissions by allocating regional aggregated data using certain socioeconomic indicators on the large scale, which means that China’s gridded emissions can easily be under- or overestimated [34]. Zhao (2012) established China’s earliest 25 × 25 km gridded CDEs dataset based on point emission source data from thermal power plants, but thus far, it has been difficult to achieve a true spatial analysis [48]. Recently, Cai (2018) has built the most comprehensive and integrated database of China’s 10 × 10 km gridded CDEs, incorporating various official data at the city level, as well as data from field surveys and visits by researchers and others. However, the three main deficiencies of the above database are fixed [34]: (1) the spatial resolution of the existing databases is relatively low; (2) the existing databases adopt mainly top-down methods, which cannot provide accurate exterior space characteristics for each grid; and (3) the existing databases cover only environmental emissions and are not linked to the socioeconomic data for each grid. Consequently, current studies of spatial gridded data still concentrate on the regional, provincial, and urban agglomeration levels, and it is difficult to delve into the internal space of urban areas in order to gain reliable research data. Due to the lack of high-precision gridded data support for CDEs, it is very important, and difficult in practice, to optimize urban spatial layouts from the perspective of CDEs. For the sustainable development of different spatial scales in China, this study selected Nanjing as a case study and used the newly emerging China high-resolution emission gridded data (CHRED) to study the spatial zoning of CDEs at the intra-city level.

The remainder of the study is arranged as follows. The next section provides information on the study area and the multiple datasets employed in this paper, as well as the theoretical framework and methodology used to analyze spatial zoning at the intra-city level. Section 3 presents the results of the optimum resolution, CDEI trends, and spatial zoning in Nanjing. In Section 4, we discuss the characteristics of different zones and the limitations of our study. Finally, Section 5 summarizes several valuable conclusions and proposes the necessary policy implications in order to achieve China’s carbon reduction targets.

## 2. Materials and Methods

### 2.1. Study Area and Dataset

#### 2.1.1. Study Area

**Figure 1:**
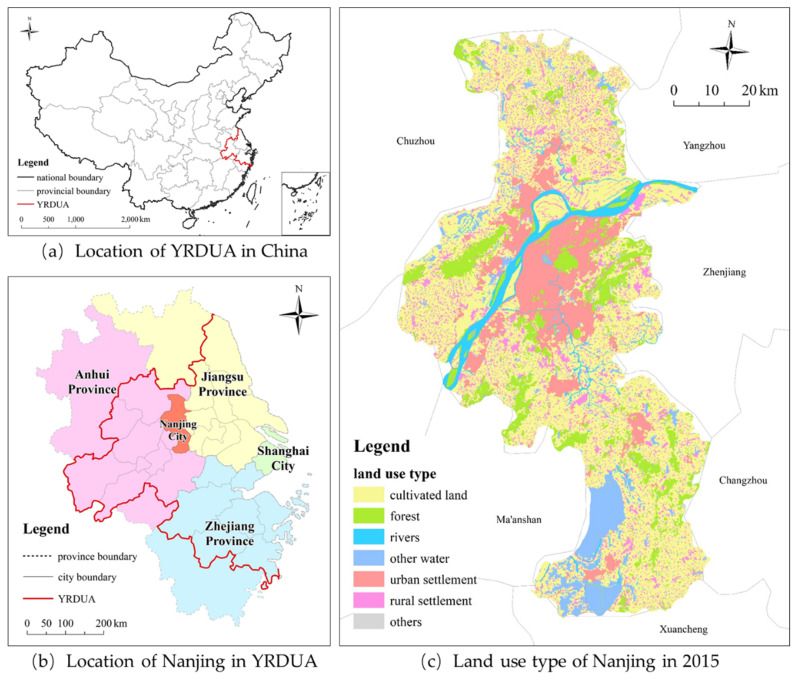
Location of and land-use types in Nanjing.

The city of Nanjing is located in the Yangtze River Delta urban agglomerations (YRDUA) in eastern China (Figure 1), a world-class urban cluster consisting of 26 prefecture-level cities covering Shanghai City and the provinces of Jiangsu, Anhui, and Zhejiang. Nanjing is one of the regional centers of China’s industrial productivity and the main industrial base in China. At the same time, it is also the provincial capital of the Jiangsu Province and a national low-carbon pilot city. By 2021, Nanjing hosted more than 9 million residents and occupied an area of 6587 km^2^, with a built-up area of 868 km^2^; thus, the urbanization rate has exceeded 85%. Its GDP and per capita GDP reached CNY 1635 billion and CNY 174,520 (25,805 USD; 1 USD = CNY 6.763), respectively, which represents a nearly threefold increase between 2011 and 2021. Such high economic growth inevitably increases CDEs due to rapid changes in urban population, vehicle stock, lifestyles, technological improvements, and production and consumption activities [49]. As the economy is expected to continue to expand in the near future, Nanjing is an ideal sample for CDEs research at the intra-city level.

#### 2.1.2. Dataset

The original data for CDEs used in this study are the CDEs data for the 10 × 10 km spatial resolution released by CHRED in 2015, which combine industrial energy consumption and processes, agriculture, service industries, urban lifestyles, rural lifestyles, and traffic emissions. According to the information provided on the official website (http://www.cityghg.com, accessed on 13 December 2022), it employs a unified data source and standardized data processing methods for establishing China’s urban CDEs dataset [50,51]. Some preconditions were followed when the dataset was applied. First, according to existing research, the distribution of CDEs is directly affected by human activities, showing a significant positive spatial correlation. Second, the CDEs data used in this study were the annual averaged data, with the short-term and severe impacts on the meteorological elements not being considered, especially within the same city. Third, due to the characteristics of fluidity and diffusivity, and ignoring the effects of wind and rainfall, there could theoretically be an attenuation phenomenon of CDEs from high-value areas to surrounding areas with increasing distance.

Supplementary data exist in addition to the CDEs data (Table 1). The annual data from the Suomi National Polar-Orbiting Partnership-Visible Infrared Imaging Radiometer Suite (NPP-VIIRS) satellite NTL remote sensing image and the China Normalized Difference Vegetation Index (NDVI) in 2015 were obtained from the China Resource and Environment Science Data Center (RESDC, https://www.resdc.cn, accessed on 23 November 2021). The land-use vector data for Jiangsu Province and the population raster data for Nanjing were obtained from the Yangtze River Delta Scientific Data Center (YRDSDC), National Earth System Scientific Data Sharing Infrastructure, National Science & Technology Infrastructure of China (http://geodata.nnu.edu.cn, accessed on 11 March 2022).

### 2.2. Theoretical Framework

The scale and intensity of CDEs are largely influenced by human economic and social activities, representing an obvious spatial heterogeneity [52]. Therefore, the structure, function, and pattern of a city all have a significant impact on CDEs. As a highly open system, there could be a huge carbon exchange between the city and its surrounding areas, and its carbon cycle process involves the vast space outside the central urban area. Generally speaking, the more economically developed a city is, the larger the exterior zone that will be affected [53].

However, existing studies have focused mainly on the CDEs from land at the macro and meso scales, and most of them have neglected the spatial match between CDEs and land utilization. The fundamental reason for this situation is that the original data for carbon emissions have been derived mainly from natural carbon fluxes and socioeconomic emissions accounting. The former are mainly fixed-point observation data, and the latter are based mostly on statistical data (generally from provincial, city, or county spatial units, with the minimum being the town or street scale), which makes it difficult to determine the spatial relationship between them. Therefore, this study attempted to solve the following two issues: (1) how to carry out a more accurate spatial matching study of CDEs at the intra-city level based on CHRED and (2) how to study the spatial zoning of CDEs at the intra-city level based on high-precision grid cells of CDEI data and their spatial differentiation characteristics.

**Figure 2:**
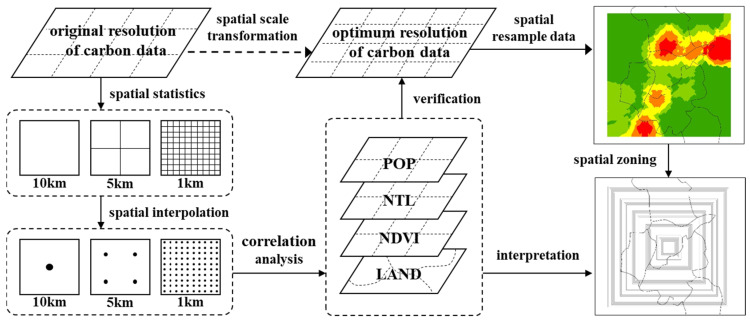
Analytical framework of the study.

The analytical framework used as the basis for this study is illustrated in Figure 2. The main processes were as follows:

Step 1: This involved spatial scale transformation of the original carbon emissions data. In terms of CDEs at the intra-city level, the original CHRED 10 km resolution data were relatively coarse, so they needed to be finely processed with spatial granularity. In light of the attenuation phenomenon associated with CDEs, this study adopted the spatial interpolation method to resample the original data in order to achieve the correct scale conversion of carbon emissions data from low to high resolution [54].

Step 2: The optimum spatial resolution grid cell was selected. The relationship between the refined grid cell data at different high resolutions obtained from the original 10 km resolution data and the supplementary data (i.e., socioeconomic data, NTL data, and land-use data) was analyzed so as to select the most suitable spatial resolution unit to carry out subsequent spatial zoning studies.

Step 3: A zoning analysis based on the variation in CDEs was conducted. Based on the optimum spatial resolution grid unit selected in Step 2, square layers depending on the grid cell were constructed in order to study the spatial zoning of Nanjing. By calculating the variation in carbon emissions data in the different layers, the spatial zoning of CDEs inside Nanjing could be explored.

### 2.3. Methods

#### 2.3.1. Spatial Interpolation

**Figure 3:**
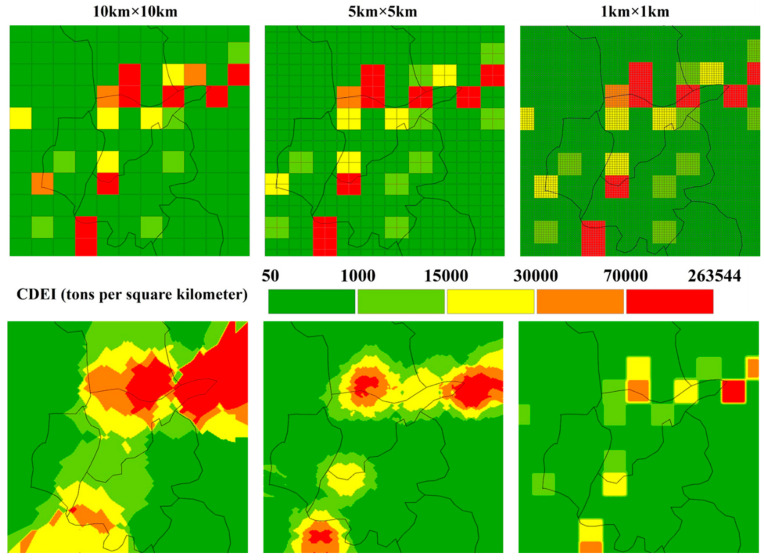
Different resolution and spatial interpolation maps of the study area.

For the study of CDEs at the intra-city level, the 10 × 10 km resolution grid was too coarse to observe the surface details, so spatial interpolation was an effective method to convert the CDEs data of the original 10 km grid into a high-resolution unit. The refined measure of the original 10 km resolution data used 1 km grid cells per segment. The CDEI value of the newly generated grid unit was the average value of the CDEI and the total area of the original 10 km grid unit, that is, the CDEI per square kilometer. On this basis, the center point of the newly generated 1 km grid cell was extracted, and the CDEI value of each grid cell was taken as the attribute value of the center point, so as to conduct a Kriging interpolation in order to obtain the spatial interpolation map. Similarly, the Kriging method was used to obtain spatial interpolation maps of 5 and 10 km grid cells (Figure 3).

#### 2.3.2. Correlation Analysis

**Figure 4:**
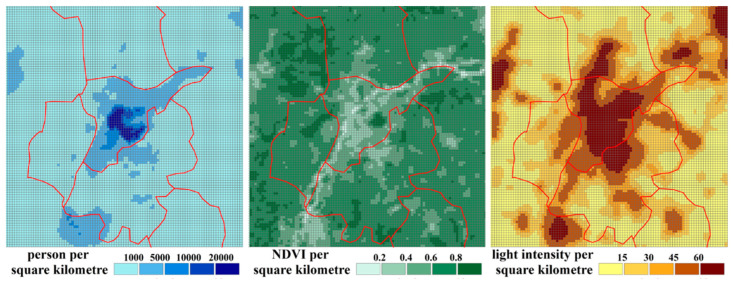
POP, NVDI, and NTL grid data.

The purpose of the above interpolation process is to refine the spatial resolution of the CDEs data, so that CDEs could be transitional in spatial distribution. Moreover, it makes the transition of CDEI between different grids smoother and more reasonable and improves the rationality of the spatial heterogeneity of CDEs following grid refinement. However, this inevitably resulted in errors between the newly resampled data and the original data. In order to select the optimal resampled data, a correlation analysis between the resampled data and the population, NTL, and NDVI data with a spatial resolution of 1 km was needed (Figure 4). Therefore, the above three different spatial interpolation maps were reconfigured into 1 km grid cells, and then the CDEI values in each grid cell were counted and verified with the population, NTL, and NDVI data of the 1 km grid to determine the best resolution of the resampled CDEs data.

Considering the strong spatial autocorrelation of the CDEs data, a spatial regression model was introduced alongside the traditional regression model. Compared to the classic ordinary least squares model (OLSM), the spatial lag model (SLM) is able to better describe and explain the related problems arising from spatial effects. The OLSM set out in this paper was as follows:

(1) $$Y=\beta_{0}+\sum_{i=1}^{m}X_{i}\beta_{i}+\varepsilon$$

where *i* represents different explanatory variables; *m* is the number of explanatory variables; *Y* is the standardized result of the dependent variable; *X_i_* is the standardized result of the explanatory variable; *β*_0_ is a constant coefficient; *β_i_* is the regression coefficient of the explanatory variables; and *ε* is the random error term.

The SLM mainly explores whether there is a diffusion phenomenon for each variable in a particular area. The SLM set out in this paper was as follows:

(2) $$Y=\rho WY+\sum_{i=1}^{m}X_{i}\beta_{i}+\varepsilon$$

where*ρ* is the spatial correlation coefficient; *W* is the spatial weight matrix, and threshold distance has been adopted in this paper; and the other variables are as defined above.

In addition to the goodness of fit *R*^2^ test, common test criteria include log likelihood (LogL), the Akaike information criterion (AIC), and the Schwartz criterion (SC). The larger the LogL, the smaller the AIC and SC, and the better the fit of the model [55]. The named indicators can be used to compare the classic linear regression models estimated by OLSM and SLM.

#### 2.3.3. Spatial Zoning

After determining the optimum resolution for resampling the data, it was necessary to reveal the attenuation phenomenon of CDEs from high-value areas to surrounding areas with increasing distance. This paper adopted as its measure the change in value of CDEI in different layers so as to summarize the spatial zoning patterns of CDEs.

**Figure 5:**
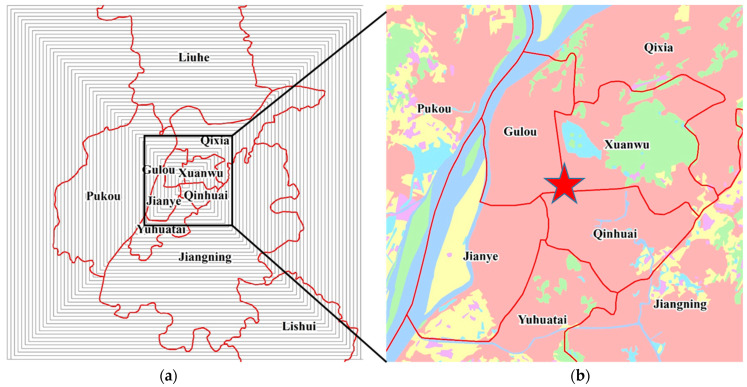
Square layers construction for Nanjing. (**a**) Square layers inside the urban area; (**b**) Central point of Nanjing.

(1) Square layers construction. We took the geometric center of the urban area as the central point and expanded outward to build square layers in turn. Therefore, the first layer is 1 km away from the center point, including four grid units; the second layer expands outward to 2 km, including 12 grid units, and so on (Figure 5).

In the square layers constructed, each layer is called an “individual layer”, and the per-unit CDEI within this layer is qc*n* (ton/km^2^). For example, the first individual layer is 1 km away from the center point, which includes four grid cells, and qc1 is the average value of the carbon emissions data for these four grid cells. The second individual layer is 2 km away from the center point, which includes 12 grid cells, and qc2 is the average value of the carbon emissions data for these 12 grid cells. By analogy, if the distance between an individual layer and the center point is *n* km, the number of grid cells within this layer is 8*n* − 4.

In addition, all layers inside this layer (including itself) are called “accumulative layers”, and the per-unit CDEI of the accumulative layer is QC*n*. According to the above, if the per-unit CDEI of the independent layer is qc1, qc2, …, qc*n*, then QC1 = qc1, QC2 = $\frac{\mathrm{qc}1\times 4+\mathrm{qc}2\times 12}{16}$, …, QC*n* = $\frac{\sum \mathrm{qc}n\times \left(8n-4\right)}{4n^{2}}$.

(2) According to the change in values of the CDEs data for each individual layer, accumulative layer, and anteroposterior layer, the relationship between the varying CDEs data and the distance to the center point was comprehensively analyzed, and the spatial zoning pattern of CDEs was proposed from the results.

## 3. Results

### 3.1. Results for the Optimum Resolution

#### 3.1.1. Spatial Cluster Analysis

**Figure 6:**
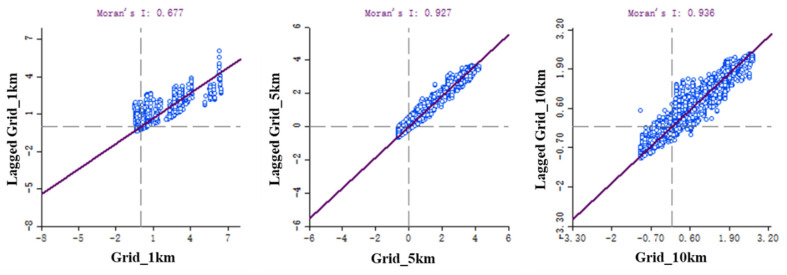
Moran’s I index for different spatial resolutions.

The spatial interpolation maps generated from the CDEs data with 1, 5, and 10 km resolutions were reassigned to 1 km grid cells to obtain three different sets of carbon emissions resampled data. The results of Moran’s I index showed that the above three sets of resampled carbon emissions data of different resolutions showed obvious spatial clustering. According to the results calculated by the GeoDa software package, (Version 1.18.0) the local Moran’s I index based on the resampled carbon emissions data with 1, 5, and 10 km resolutions were 0.677, 0.927, and 0.936, respectively (Figure 6), all of which showed positive spatial correlations, indicating that the three sets of resampled carbon emissions data showed spatial agglomeration. Therefore, a correlation analysis between the resampled carbon emissions data (1 km grid cells) and the population, NTL, and NDVI data (1 km grid cells) was carried out in order to determine the optimum resampled data for the three sets, which allowed the SLM to be utilized along with the traditional OLSM.

#### 3.1.2. Correlation Analysis

The GeoDa software package was used to test and estimate the OLSM and SLM. The fitting results are presented in Table 2. The goodness of fit (*R*^2^) test values for SLM for each resolution (1, 5, and 10 km) are obviously higher than those for the OLSM. When further comparing LogL, AIC, and SC, the results show that the LogL values for SLM are higher than those for OLSM, while the AIC and SC values for SLM are lower than those for OLSM for each resolution. It is clear that the SLM is superior to the classic OLSM.

The estimated results for OLSM and SLM are listed in Table 3 and Table 4. As can be seen from Table 3, three variables—population size (POP), NDVI, and NTL intensity—all passed the significance test at the 1% and 5% levels (probabilities equal 0.01, 0.05, and 0.10 at the 1%, 5%, and 10% mean significance levels, respectively). However, as can be seen from Table 4, the resampled carbon emissions data at 5 km resolution appear to be better. First, of the three different resolutions, only the correlation coefficients of all three variables (POP, NDVI, and NTL) for the 5 km resolution passed the significance test at the 1% level. POP for the 1 km resolution and NDVI for the 10 km resolution failed to pass the significance test at the 10% level. Second, the estimated results showed that the regression coefficients of the variables at the 1 and 5 km resolutions were more reasonable for both OLSM and SLM. This is because the regression coefficient of NDVI was negative for both the 1 and 5 km resolutions, indicating that CDEI was negatively correlated with NDVI, which is consistent with the view that vegetation has been considered a “carbon sink” in previous studies [56]. In other words, errors occurred in the resampled carbon emissions data for the 10 km resolution, which should not be considered. In summary, according to the results of the regression analysis, this study considers the 5 km resolution to be the optimum one, and so the spatial zoning analyses, based on the resampled carbon emissions data, were carried out employing this resolution.

### 3.2. Results for qcn and QCn

#### 3.2.1. Individual Layers

**Figure 7:**
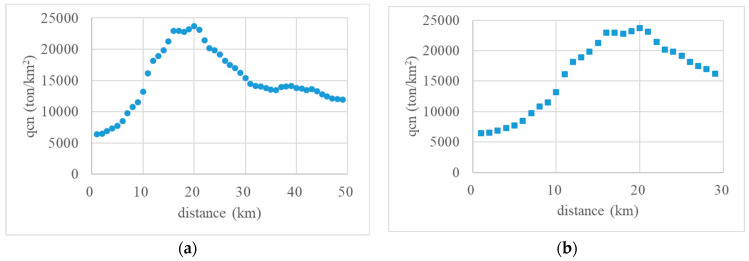
Statistical results for individual layers in Nanjing. (**a**) Whole urban area; (**b**) Central urban area.

After the 5 km resolution resampled carbon emissions data were assigned to the 1 km grid cell, we generated square individual layers covering the Nanjing urban area. The variation in the CDEI of layers based on their distance to the central point is shown in Figure 7a. The horizontal axis represents the distance of individual layers to the central point of Nanjing, and the vertical axis represents the per-unit CDEI within each layer (qc*n*). Obviously, for distances of 0–7 km, the CDEI was low, showing a smooth variation; for distances of 7–16 km, the CDEI increased rapidly; for distances of 16–21 km, the CDEI reached a peak value and maintained this high value; for distances of 21–31 km, the CDEI decreased rapidly; and for distances of more than 39 km, the CDEI began to decrease and tended to be stable.

Figure 7b illustrates more clearly the variation in the CDEI of individual layers in the central urban area. For distances of 0–7 km, the CDEI increased steadily from a low value; for distances of 7–16 km, the CDEI increased significantly; for distances of 16–21 km, the CDEI remained high and stable; and for distances of more than 21 km, the CDEI began to decrease. This demonstrated that the range of 16–21 km from the central point of Nanjing was the area of the greatest concentration of carbon sources in the city.

#### 3.2.2. Accumulative Layer

**Figure 8:**
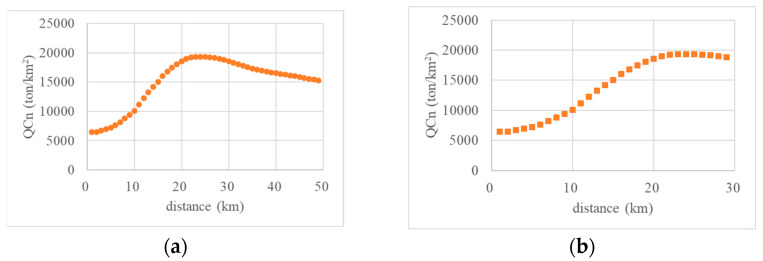
Statistical results for accumulative layers in Nanjing. (**a**) Whole urban area; (**b**) Central urban area.

The variation in per-unit CDEI of accumulative layers (QC*n*) based on their distance to the central point is shown in Figure 8a. In general, with the continuous expansion of layers, the CDEI in Nanjing first increased and then gradually decreased. For distances to the central point of less than 12 km, the CDEI increased rapidly; for distances from the center point of approximately 24 km, the CDEI grew slowly and gradually reached a peak; and for distances of approximately 28 km, the CDEI remained high and changed little; thereafter, the CDEI decreased quite slowly.

Figure 8b reflects the variation in the CDEI of accumulative layers in the central urban area of Nanjing. For distances of less than 24 km from the central point, the CDEI increased for this entire distance, and only began to decrease once the distance threshold had been exceeded.

### 3.3. Results for Spatial Zoning

#### 3.3.1. Variation in CDEI

To further demonstrate the effect of layers spread on CDEI, this paper also analyzed changes in QC*n*. Starting from the second accumulative layer, the decrease in the value of the CDEI was QC1−QC2, QC2 − QC3, …, QC(*n* − 1)−QC*n*, that is, the difference between the CDEI of the front and back accumulative layers.

**Figure 9:**
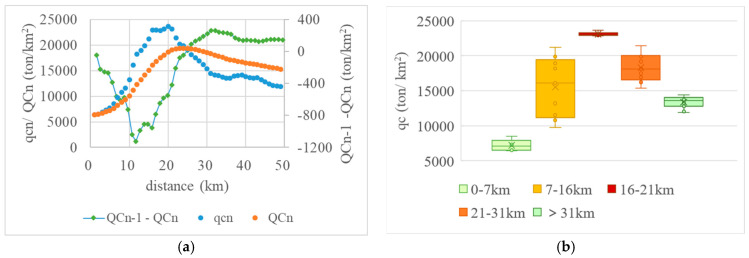
Variation in CDEI for Nanjing. (**a**) Scatter plot of CDEI; (**b**) Box plot of CDEI.

As shown in Figure 9a, the first inflection point appears at a distance of 12 km away from the center point. Within this range, the decrease in value is negative and continues to decrease as the layer spreads outward, indicating that CDEI is increasing ever more rapidly. The second inflection point occurs at a distance of 32 km, that is, in the range 12–32 km, within which the rate of decrease increases continuously and changes from negative to positive for the first time at a distance of 25 km. Beyond a distance of 32 km, the rate of decrease reduces slowly but remains positive as the layer continues to spread outward, indicating that CDEI begins to decline and then gradually slows down.

Based on the variation in CDEI shown in Figure 7a, Figure 8a, and Figure 9a, we then used a box plot to divide the values into groups (see Figure 9b). The layers extending to the center point below 7 km were categorized as the first group, and the CDEI values in this group were low, with an average value of only 7215 ton/km^2^. Layers extending to the center point in the range 7–16 km were categorized as the second group, and the CDEI values in this group increased rapidly, with an average value of 15,507 ton/km^2^. Layers extending to the center point in the range 16–21 km were categorized as the third group, and the CDEI values in this group surged to a peak, with an average value of 23,099 ton/km^2^. Layers extending to the center point in the range 21–31 km were categorized as the fourth group, and the CDEI values in this group decreased significantly, with an average value falling to 18,307 ton/km^2^. Layers extending to the center point beyond 31 km were categorized as the fifth and final group, and though the CDEI values in this group continued to decline, they reached a stable end value, with an average value of 13,393 ton/km^2^.

#### 3.3.2. Spatial Zoning in Nanjing

Based on the variations in CDEI described above, this study categorized the spatial zoning patterns of CDEs in Nanjing (Table 5). For distances of less than 7 km from the center of the square layers, this formed the central budding zone. The CDEI value of this zone was low, and the average value was only 31.24% of the average level of the peak group. However, the growth rate of CDEI in this zone had already begun to increase, with a rate of approximately 5.73%. For distances of 7–16 km from the center, this formed the interior burgeoning zone. The growth rate of CDEI was more rapid here, reaching a rate of 8.11%, and the average CDEI value in this zone reached 67.13% of the average level of the peak group. For distances of 16–21 km from the center, this formed the urban peak zone. The CDEI value reached a peak and remained stable, with a growth rate of only 0.10%. For distances of 21–31 km from the center, this formed the suburban recessionary zone. The variation in CDEI values in this zone began to decline after experiencing a peak, at a rate of about −2.50%, and the average CDEI value in this zone dropped to 79.26% of the average level of the peak group. For distances beyond 31 km from the center, this formed the exterior balanced zone. The variation in CDEI values declined further, but the rate of reduction was small because the CDEI value basically tended to be stable, remaining at about 57.98% of the average level of the peak group.

**Figure 10:**
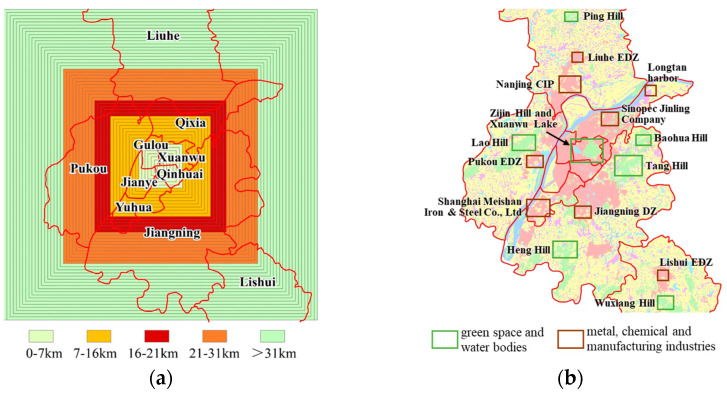
Maps of spatial zoning of Nanjing. (**a**) Spatial zoning of layers; (**b**) Ecological and industrial areas.

Figure 10a clearly reflects the layout of spatial zoning of CDEs inside Nanjing. The central budding zone occupies the four core districts (FCD) of Gulou, Xuanwu, Qinhuai, and Jianye and is the core area where more than half of Nanjing’s urban residents live and work. Although this area is dominated by urban built-up areas, it also protects large green spaces and water bodies, such as Zijin Hill and Xuanwu Lake. Because these open spaces could reduce the emission efficiency related to the urban heat island effect [57], this ensured that the CDEI value was relatively low. This also validated the results of the regression model, that is, CDEs had a small, or even a negative, correlation with the variable POP. This showed that, although the population of FCD is dense, CDEs were more affected by energy consumption and industrial processes.

The distribution characteristics of the interior burgeoning zone, urban peak zone, and suburban recessionary zone also supported this result. Extending outward from the FCD, a large number of steel, petrochemical, electric power industries, and economic development zones (EDZ) dominated by manufacturing industry are located, which are the areas of carbon source concentration in Nanjing. This area includes the Sinopec Jinling Company and Longtan harbor along the Yangtze River in Qixia District, Nanjing Chemical Industry Park (CIP), and Liuhe EDZ in Liuhe District, which are located to the north of the FCD. It also includes Shanghai Meishan Iron & Steel Co., Ltd. (Nanjing, China), Jiangning Development Zone, and Lishui EDZ, which are located in the south of the FCD. Pukou EDZ is located in west of the FCD (Figure 10b). In addition, a large number of new satellite towns have been built in these areas. In general, the greater the distance from the city center, the higher the proportion of transport energy consumption [58]. This leads to energy use for transport that is greater in the satellite towns than in the FCD. However, as a city where hills are a natural feature, the extent of hill volume in the suburban recessionary zone has gradually increased in Nanjing, including the hills of Lao, Heng, Baohua, Tang, etc., all playing an important role as carbon sinks. This is also the main reason for the decline in CDEI in this zone compared to the urban peak zone.

The exterior balanced zone is composed mainly of a large area of cultivated land and a large number of scattered rural settlements, which are used mainly to ensure food security in the city. Since chemical fertilizers, agricultural machinery, and pesticides are widely used in agricultural production [59], there is also a certain degree of carbon emissions originating from cultivated land. At the same time, ecological green spaces, such as Ping Hill and Wuxiang Hill, at the periphery also play a role in carbon reduction, so that the CDEI in this zone has basically reached a stable state while generally declining.

## 4. Discussion

Obviously, the industrial energy consumption sector is the largest contributor to CDEs in Nanjing. As previous studies have revealed, carbon emissions from industrial processes relate mainly to emissions from the chemical or physical transformation of materials during industrial production. The four major industries, namely, the automobile industry, steel production, electronic components manufacturing, and petrochemical materials, have become large and comprehensive emitters, accounting for more than 90% of total energy consumption, and emphasize that Nanjing has insufficient diversity in its energy consumption structure and a high dependence on the consumption of traditional energy sources [60]. According to the statistics, by the year 2016, the petrochemical industry was responsible for releasing 86.39 million tons of CDEs, which accounted for 82% of total industrial CDEs [61]. Although the petrochemical industry is considered to be a highly polluting emitter of CDEs, it plays a key role in the process of industrialization-based urbanization in Nanjing through its important function of providing employment and integrating various industries [62]. In recent years, however, Nanjing has promoted the upgrading of the heavy chemicals industry and of innovations in production technology and processes. Nevertheless, Nanjing is also facing constraints related to investment, production costs, and shrinking markets, so there is great pressure to ensure energy conservation and emissions reduction in traditional industries.

Moreover, the relatively low levels of CDEI in the FCD indicate that the carbon emissions of more compact urban districts—that is, built-up areas characterized by relatively high densities, mixed land use, and pedestrian-oriented habitation patterns—are lower than those of dispersed districts [13]. Similar phenomena have been observed in many developed cities in Italy [63], Canada [64], and Japan [11]. Many researchers believe that the development of urban spatial patterns involving the spatial evolution and replacement of various socioeconomic elements, including economic activities, population, urban land use, and various public facilities, could affect the generation and diffusion of CDEs to a great extent [65]. For example, a compact urban spatial development pattern could efficiently shorten the distance and time taken by people to commute to work, making them more inclined to use public transportation, and thereby reducing CDEs [66]. In addition, urban green ecological spaces are also crucial for carbon absorption, through photosynthesis by vegetation, particularly in the central budding and exterior balanced zones.

As a microcosm of YRDUA, the results of studies on Nanjing show that urban agglomerations are highly concentrated areas of population and economic activities, which inevitably lead to environmental pollution and CDEs driven by rapid industrialization and urbanization [67]. Such problems of environmental pollution characterized by CDEs have become a major obstacle to high-quality regional development. This means that carbon emissions are not only an environmental problem but also a development problem, especially for developing countries that are in the process of industrialization and urbanization. The Chinese government has pledged that carbon emissions will peak around 2030 and then begin to decline according to the “dual carbon” target. Combined with the research conclusions of this paper, we believe that it is necessary to emphasize the importance of spatial layout optimization even further in order to achieve the low-carbon economy that China is striving to reach.

The Chinese government’s newly established territorial spatial planning strategy is one of the most important land-use and urban layout strategies currently in use in China. Its benefits could be realized through a process of identifying and delimiting important ecological, agricultural, and urban areas using integrated, detailed, and special master plans at the national, provincial, city, county, and township levels [68]. That is to say, territorial spatial planning can adequately determine future land use through adjustments to land-use types, land-use intensity, and spatial distribution. Targeting the aim of carbon reduction, specifically, the formulation and implementation of territorial spatial planning at the city level should help to explore the relationship between economic development of different county units and CDEs within cities. Combined with the characteristics of the different modes and stages of urban development, targeted and differentiated low-carbon development strategies should also be proposed. In particular, an index system composed of total CDEs, carbon aggregates, CDEI, carbon sink intensity, etc., needs to be established and then converted to spatial planning elements to promote differentiated carbon emissions reduction goals, methods, and countermeasures to achieve sustainable low-carbon spatial planning guidance. Strategies such as carbon trading and carbon compensation, initially proposed by developed countries to cope with climate change in their planning practices, are thus worthy of study in order to guide planning in the Chinese context [69,70].

## 5. Conclusions

This study aimed to analyze how to recognize the spatial heterogeneity of CDEs at the intra-city level, and therefore reveal spatial zoning. Specifically, based on the CHRED published in 2015, as well as gridded data for NTL, NDVI, and population, the contribution of this paper is the proposal of a theoretical framework to solve two fundamental issues, namely, (1) how to study the distribution of CDEs inside an urban area based on CHRED, with relatively coarse spatial resolution, and (2) how to summarize the spatial pattern of CDEs based on CEI trends and their spatial differentiation characteristics. Moreover, a spatial interpolation method was used to convert the CDEs data for the original 10 km grid into high-resolution resampled data; OLSM and SLM were then employed as models to select the optimal resampled data according to the results of correlation analysis. Third, multiple square layers surrounding the central portion of Nanjing and covering the entire city were constructed in order to explore the variations in CEI along different layers from inside to outside. Hence, the spatial zoning of CDEs in Nanjing was summarized according to the value and rate of variation of CEI in the different layers, as well as the layers’ distances to the center point of the city.

Overall, compared with previous studies, our study has revealed the spatial zoning of CDEs inside Nanjing City for the first time. Several valuable conclusions were reached through these analyses. In terms of variation, the CEDIs of the five zones showed an inverted “U-shaped” trend that first increased, then decreased, and finally stabilized. In terms of spatial pattern, this inverted “U-shaped” trend perfectly echoed what we mentioned in the theoretical framework, the scale and intensity of CDEs are largely influenced by human economic and social activities and the structure, function, and pattern of a city have significant impacts on CDEs. The findings are basically consistent with the existing studies of the differences in CDEs between urban and suburban areas in large cities, such as Tianjin [71], New York [72], and Toronto [73]. In fact, it is generally accepted that urban areas have lower per capita CDEs levels than surrounding areas due to high population density, high energy efficiency, centralized energy supply, and relatively sound public transportation systems. In addition, zoning divisions based on CDEI values and their variations are partly inspired by related topics, such as carbon neutral zoning [74] and carbon offset zoning [75], and the results of this paper can also provide support for the formulation of collaborative CDEs reduction schemes at the intra-city level.

Although we found the reasonable explanation for spatial zoning in Nanjing and highlighted that the industrial energy consumption sector is the largest contributor to CDEs, following a period of continuous urbanization and industrialization, the energy consumption sector, represented by the petrochemical industry, is still one of the most important sources of CDEs in the city. In the foreseeable future, the volume of CDEs will continue to increase more significantly than the volume of carbon sequestration, which needs to be balanced by a larger external ecosystem. It is clear that, as the area of concentrated carbon sources in the city continues to increase, the CDEI of the interior burgeoning zone, the urban peak zone, and the suburban recessionary zone will also continue to increase. At the same time, its area of coverage will also continue to grow, shrinking the existing exterior balanced zone. Industrial transformation is a possible means to resolve the problem. The emerging information and communications technology (ICT) industry has the potential to reduce carbon emissions [76]; therefore, Chinese megacities such as Nanjing must make strategic decisions about how their ICT industry should grow and expand.

Evidently, the limitations of this study should be mentioned and need to be addressed in future studies.

(1) There are obvious scale issues in the study of carbon sources and carbon sinks in terrestrial ecosystems, and this paper takes Nanjing as a sealed area, without considering the carbon cycle relationship with terrestrial ecosystems in a larger spatial range outside Nanjing.
(2) The resampled data for the spatial zoning division were derived from the 10 km spatial resolution of CHRED, and this study has refined the resolution problem by spatial interpolation in order to achieve spatial scale transformation. Therefore, the resampled data can only reveal the spatial heterogeneity characteristics of Nanjing’s CDEs from the overall spatial pattern; they cannot accurately match the grid cells in the same locations.
(3) With the development of urbanization and industrialization in Nanjing, CDEs are still in the process of dynamic change. Restricted by the difficulty of data collection and matching, this paper obtained only basic data from 2015, because of which it is impossible to carry out an empirical analysis of the annual changes in the spatial zoning of CDEI. However, combined with the increasing population and fossil energy consumption in Nanjing and the slow transformation of the industrial structure, the scale of CDEs and their scope of influence arising from human activities and energy consumption will continue to increase, the CDEI of the interior burgeoning zone, the urban peak zone, and the suburban recessionary zone will also continue to increase, and its coverage will also grow and continue to extend its range.

## Figures and Tables

**Table 1:** Data and their sources employed in this study.

Data Type	Name	Source
Carbon emissions data	China High-Resolution Emission Gridded Data	CHRED
Night-time light (NTL) data	National NPP-VIIRS satellite night-time light remote sensing image	RESDC
Land-use data	NDVI gridded data of YRDUA	
	Land-use vector data for Jiangsu Province	YRDSDC
Socioeconomic data	Population gridded data for Nanjing City	

**Table 2:** Statistical test results for the SLM and OLSM models.

Test Item	1 km Resolution		5 km Resolution		10 km Resolution	
	*OLSM*	*SLM*	*OLSM*	*SLM*	*OLSM*	*SLM*
*R* ^2^	0.120	0.786	0.214	0.978	0.156	0.963
LogL	−124,185	−117,005	−108,645	−91,716	−110,645	−94,928
AIC	248,378	234,020	217,297	183,442	221,299	189,866
SC	248,407	234,057	217,326	183,478	221,327	189,902

**Table 3:** Estimated results for the OLSM.

Variable	1 km Resolution		5 km Resolution		10 km Resolution	
	Coef.	Prob.	Coef.	Prob.	Coef.	Prob.
CONSTANT	11,625.600	0.000	17,939.000	0.000	5827.590	0.000
POP	−1.474	0.000	−1.235	0.000	−0.280	0.000
NDVI	−19,863.200	0.000	−21,359.700	0.000	2090.850	0.013
NTL	643.268	0.000	461.596	0.000	290.865	0.000

**Table 4:** Estimated results for the SLM.

Variable	1 km Resolution		5 km Resolution		10 km Resolution	
	Coef.	Prob.	Coef.	Prob.	Coef.	Prob.
W_grid	1.000	0.000	1.000	0.000	1.000	0.000
CONSTANT	1696.590	0.081	392.377	0.168	−201.468	0.152
POP	−0.072	0.362	−0.103	0.000	−0.039	0.001
NDVI	−4259.420	0.001	−1336.320	0.000	125.088	0.483
NTL	40.881	0.000	21.014	0.000	5.680	0.000

**Table 5:** Results for the spatial zoning patterns in Nanjing.

Distance (km)	Value and Trend	Rate of Change	Spatial Zoning
0–7	low value, increasing	slow growth	central budding zone
7–16	middle value, increasing	rapid growth	interior burgeoning zone
16–21	high value, stable	steady	urban peak zone
21–31	middle to high value, decreasing	intermediate reduction	suburban recessionary zone
>31	middle value, decreasing	very slow reduction	exterior balanced zone

## Data Availability

The data presented in this study are available on request from the corresponding author. The data are not publicly available due to restrictions.

## List of Abbreviations

CDEs	Carbon dioxide emissions
CHRED	China high-resolution emission gridded data
CDEI	Carbon dioxide emissions intensity
GDP	Gross domestic product
EDGAR	Emissions Database for Global Atmospheric Research
IPCC	Intergovernmental Panel on Climate Change
NTL	Night-time light
DMSP-OLS	Defense Meteorological Satellite Program-Operational Line scan System
YRDUA	Yangtze River Delta urban agglomerations
NPP-VIIRS	National Polar-Orbiting Partnership-Visible Infrared Imaging Radiometer Suite
NDVI	Normalized Difference Vegetation Index
YRDSDC	Yangtze River Delta Scientific Data Center
OLSM	Ordinary least squares model
SLM	Spatial lag model
LogL	Log likelihood
AIC	Akaike information criterion
SC	Schwartz criterion
FCD	Four core districts
EDZ	Economic development zones
CIP	Nanjing Chemical Industry Park
